# Hydrogen Sulfide Attenuates β2-Microglobulin-Induced Cognitive Dysfunction: Involving Recovery of Hippocampal Autophagic Flux

**DOI:** 10.3389/fnbeh.2019.00244

**Published:** 2019-10-25

**Authors:** Si-Min Chen, Yi-Li Yi, Dan Zeng, Yi-Yun Tang, Xuan Kang, Ping Zhang, Wei Zou, Xiao-Qing Tang

**Affiliations:** ^1^Department of Neurology, The First Affiliated Hospital, University of South China, Hengyang, China; ^2^Department of Neurology, The Affiliated Nanhua Hospital, University of South China, Hengyang, China; ^3^Institute of Neuroscience, Hengyang Medical College, University of South China, Hengyang, China

**Keywords:** hydrogen sulfide, β2-microglobulin, cognitive dysfunction, autophagic flux, hippocampus

## Abstract

**Background and Aim:**

Accumulation of β_2_-microglobulin (B2M), a systemic pro-aging factor, regulates negatively cognitive function. Hydrogen sulfide (H_2_S), a novel gas signaling molecule, exerts protection against cognitive dysfunction. Therefore, the present work was designed to explore whether H_2_S attenuates cognitive dysfunction induced by B2M and the underlying mechanism.

**Materials and Methods:**

The cognitive function of rats was assessed by Y-maze, Novel object recognition (NOR), and Morris water maze (MWM) tests. The levels of autophagosome and autolysosome in hippocampus were observed by transmission electron microscopy. The expression of p62 protein in hippocampus was detected by western blot analysis.

**Results:**

NaHS (a donor of H_2_S) significantly alleviated cognitive impairments in the B2M-exposed rats tested by Y-maze test, NOR test and MWM test. Furthermore, NaHS recovered autophagic flux in the hippocampus of B2M-exposed rats, as evidenced by decreases in the ratio of autophagosome to autolysosome and the expression of p62 protein in the hippocampus.

**Conclusion:**

In summary, these data indicated that H_2_S attenuates B2M-induced cognitive dysfunction, involving in recovery of the blocked autophagic flux in the hippocampus, and suggested that H_2_S may be a novel approach to prevent B2M-induced cognitive dysfunction.

## Introduction

β2-microglobulin (B2M), a component of major histocompatibility complex class 1 (MHC I) molecules, acts independently on its canonical immune function to regulate normal brain development, synaptic plasticity and related behaviors (Zijlstra et al., 1990; Huh et al., 2000; Jennifer et al., 2003; Boulanger and Shatz, 2004; Goddard et al., 2007; Shatz, 2009; Glynn et al., 2011; Hanmi et al., 2014). It is confirmed that systemic or local administration of B2M into hippocampus impairs the function of learning and memory, and there is a possibility to reverse B2M-induced cognitive defects when the excessive B2M is removed (Smith et al., 2015). The level of B2M is elevated in aging process (Yang et al., 2017), Alzheimer’s disease (AD) and HIV-associated dementia (Mcarthur et al., 1992; Brew et al., 1996; Carrette et al., 2003). In addition, increasing works have demonstrated that B2M is an independent risk factor for AD (Villeda et al., 2011; Lida et al., 2014). Thus, it is necessary for us to find approaches to antagonize B2M-induced cognitive dysfunction, which lead to the development of new strategies for treatment of AD.

Hydrogen sulfide (H_2_S), as the third gaseous signaling molecule (Łowicka and Beltowski, 2007; Szabo, 2007), plays an important role in learning and memory function (Whiteman et al., 2011). It has been demonstrated that H_2_S promotes the formation of hippocampal long-term potentiation (LTP) (Bliss and Collingridge, 1993; Abe and Kimura, 1996; Kimura, 2000), and attenuates cognitive deficits induced by lipopolysaccharide or beta-amyloid (Gong et al., 2011; Liu et al., 2015, 2016). Similarly, we have previously reported that H_2_S reverses homocysteine-induced impairments in learning and memory (Li et al., 2014, 2016). In the present work, we explored whether H_2_S ameliorates cognitive dysfunction caused by B2M in the rodent model.

Autophagy occurs as a cellular response to both extracellular stress conditions and intracellular signals and is essential for cellular growth and survival (Feng et al., 2018). The dynamic process of autophagy, termed “autophagic flux,” involves the rearrangement of subcellular membranes to sequester cytoplasm and organelles, which are delivered to the lysosome or vacuole to degrade and recycle (Levine and Kroemer, 2008). It has been proved that autophagic flux is closely related to presynaptic morphology, postsynaptic morphology and neuronal remodeling (Liang and Sigrist, 2018), associated with the enhancement of memory. The inhibition of autophagic flux was observed in mice with cognitive deficits, while activation of autophagic flux rescues these impairments of synaptic plasticity and cognition (Hara et al., 2006; Nikoletopoulou et al., 2017; Yan et al., 2018). Moreover, activating autophagy also alleviated cognitive dysfunction caused by chronic unpredictable mild stress and morphine (Gu et al., 2014; Pan et al., 2016). These findings suggest a potential protective role of autophagic flux in cognitive function. Interestingly, H_2_S regulates endoplasmic reticulum stress-mediated autophagy and autophagic neuronal cell death (Xie et al., 2017), which confirms the positive effect of H_2_S on regulating autophagic flux. Therefore, we evaluated whether H_2_S modulates the autophagic flux in the hippocampus of B2M-exposed rats.

In the present work, we have demonstrated that H_2_S ameliorates cognitive dysfunction induced by B2M and reverses the inhibition of autophagic flux in the hippocampus of B2M-exposed rats. These data suggested that H_2_S has the potential to ameliorate cognitive dysfunction induced by B2M, and may provide a new target for prevention of AD.

## Materials and Methods

### Experimental Schedule

**FIGURE 1:**
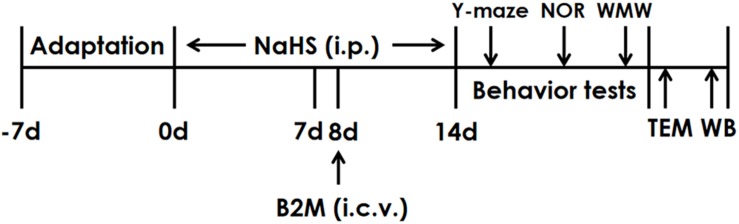
The schematic diagram of the experimental schedule. Y-maze, Y-maze test; NOR, Novel object recognition test; MWM, Morris water maze test; TEM, Transmission electron microscopy; WB, Western blot.

The rats were pretreated with NaHS for 7 days and B2M was co-treated in the 8th day. After continuously treated with NaHS for 2 weeks, all rats were subjected to behavior tests. Finally, the hippocampal autophagy levels of rats were detected by Transmission electron microscopy (TEM) and Western blot (WB) (Figure 1).

### Animals

Adult male Sprague-Dawley (SD) rats (250–300 g) were obtained from the Hunan SJA Laboratory Animal Center (Changsha, Hunan, China). They were housed individually with free access to food and water under a normal 12 h light/dark schedule (lights on at 7:00 a.m.). Housing temperature was maintained at (22 ± 2)°C and relative humidity of 55 ± 5%. Before the beginning of the experiments, rats were allowed 7 days to adapt themselves to the housing conditions. According to the National Institutes of Health Guide for the Care and Use of Laboratory Animals, all the procedures were strictly implemented and approved by the Animal Use and Protection Committee of University of South China.

### Reagents

Sodium hydrosulfide (NaHS, a donor of H_2_S) and β2-microglobulin were purchased from Sigma (Sigma, St. Louis, MO, United States). Anti-p62 antibody was purchased from Cell signaling Technology (Boston, MA, United States). Beta-actin antibody was purchased from Proteintech (Danvers, MA, United States).

### Drug Treatment Protocols

A total of 1.68 mg or 5.6 mg of NaHS was dissolved in 1 mL of phosphate-buffered saline (PBS) to equal concentrations of NaHS 30 or 100 μmol/mL, respectively. A total of 0.3 μg of B2M was diluted in 3 μL PBS and intracerebroventricularly injected (i.c.v.) at a dose of 3 μL.

### Lateral Ventricle Cannulation

Rats were firstly anesthetized with sodium pentobarbital (60 mg/kg, i.p., Sigma, St. Louis, MO, United States) and then placed in stereotaxic apparatus for operation. Using the coordinates (AP, −1.0 mm; ML, + 2 mm; DV, + 3 mm), a permanent guide cannula was aseptically and stereotaxically implanted into the right lateral ventricle of rats. After stereotaxic surgery, home-cage behaviors and wound healing of rats were monitored. It was an inclusion criterion that body weight recovered to no less than 90% of presurgery amount within 7 days.

### Intracerebroventricular Injection

Under the control of micropump, 3 μl PBS and 3 μl B2M (0.1 μg/μl) were respectively injected into the lateral ventricle with an injection rate of 0.5 μl per min. In order to make sure that the entire injection had been delivered, the injection cannula was allowed to remain for additional 5 min before removed.

### Y-Maze Test

The Y-maze is a Y-shaped platform that consists of three arms with 50 cm length and 10 cm width. And each arm was surrounded on three sides by 40 cm high walls. The three arms are connected in open ends centrally and positioned radially at 120° angles (Stoelting Co., Wood Dale, IL, United States). Each rat was placed at the center of the maze firstly and then allowed to explore freely for 5 min. Movement was recorded by an overhead camera and analyzed by the Any-maze software (SD Instruments, San Diego, CA, United States). Sequences of entering arm and numbers of entering each arm were recorded, respectively. Memory was defined by the frequency of alternately exploring different arms and was determined using the formula: total number of alternations/(total number of arm entries−2) × 100%.

### Novel Objects Recognition (NOR) Test

The NOR test is consisted of three parts: adaptation, training and testing. Two days before training, rats were adapted to the arena (50 cm × 50 cm × 60 cm) for 5 min once a day without objects. During the training part, two different objects (A and B) were placed in the testing arena. Each rat was allowed to explore the objects for 5 min. The rat was considered to be exploring the object when its head was facing the object, or the rat was touching or sniffing the object. The total time spent exploring each object was recorded by same trained observer blind to treatments, and expressed as percentage of total exploration time. In the testing part, one identical and one novel objects (A and C) were used. A rat was allowed to explore the objects for 5 min, and the time spent exploring each object was recorded. The discrimination index = [(novel object−familiar object)/(novel object + familiar object)] × 100%, was used to measure the cognitive function of rats.

### Morris Water Maze (MWM) Test

The water maze was a circular pool with diameter 180 cm and height 60 cm. The water temperature (23 ± 2)°C, light intensity, external cues in the room, and water opacity were rigorously reproduced. A transparent Plexiglas non-slippery platform (diameter 12.5 cm) was immersed under the water surface (2 cm) during acquisition phase. By a camera capture and video track software, swimming track of each rat was recorded and analyzed. The pool was divided by the software into four quadrants. As the distal spatial extra-maze cues for the rats to find the platform, several landmarks were fixed to the walls of the water maze room.

#### Acquisition Phase

The place navigation training consisted of four times of swimming per day for 5 days, and four start positions were randomly selected. And each rat was allowed a 120 s-swim to find the platform. Once the rat reached the platform, it was left 20 s on the platform, but if a rat did not reach the platform within 120 s, it was guided to the platform to remain for 20 s. The path and the escape latencies were recorded by an MT-200 Morris image motion system (Chengdu Taimeng Technology & Market Co., Chengdu, China).

#### Probe Phase

The probe test was performed after 24 h of the last swim on the 5th day. The platform was removed and each rat was allowed a free 120 s-swim. Rats were placed into the pool from another quadrant, which was opposite to the target quadrant, the former platform quadrant. The percentage of time spent in the target quadrant and the number of times that the same rat crossed the quadrant were determined.

#### Visible Platform Test

After the probe phase, visual, motor, and motivation skills were also tested with a visible platform test to eliminate the possible deficits of rats in sensorimotor processes. The platform was raised 2 cm above the water surface. The platform was moved to a novel quadrant in the pool at a fixed location for the four consisted trials, and the latency to reach the platform and the average speed were recorded.

### Tissue Sampling

The hippocampal tissue is grinded and shredded in the tissue grinder after adding ice physiological water (nine times the weight of tissue). Then, the cell lysate and PMSF are added according to the proportion of 100:1, and fully lapped to 30 min. Finally, the liquid in the grinder is transferred into the EP tube and put into 4°C centrifuged for 10 min. The supernatant fluid was separated and packed. The BCA protein quantitative kit was quantified and stored at −20°C.

### Western Blot Analysis for p62 Protein Expression

According to the molecular weight of p62, equivalent amount of proteins were run on SDS–PAGE gel (12% for p62). The electrophoresis was carried out with the constant pressure of 80 V. When the protein in the lanes ran to the junction point of the concentrating and the separating glue, the voltage was transformed into 120 V at constant pressure to continue the electrophoresis, and stopped until it ran to the bottom. According to the molecular weight of target protein, suitable size gel was cut. Then, “sandwich” (filter paper + glue + PVDF membrane + filter paper), wet transfer method constant flow membrane (300 mA, 30 min), the PVDF membrane is cut into the appropriate size and soaked in absolute methanol. After transferred to a PVDF membrane, the PVDF membrane was blocked using TBST buffer (50 mM Tris–HCl, pH 7.5, 150 mM NaCl, 0.05% Tween-20) containing 5% non-fat milk at room temperature for 2 h. Then the PVDF membrane was incubated by primary antibody of the target protein and internal reference protein (anti-p62, 1:1000; anti-beta-actin, 1:2000) overnight at 4°C. After the PVDF membrane was washed three times (10 min each time) with TBST, secondary antibody (1:5000) with 5% non-fat milk were added to incubate the PVDF membrane 2 h, and then washed for three times (10 min each time). Finally, the expression of protein p62 was analyzed by Image J on the gel imager.

### Transmission Electron Microscopy Analysis for Hippocampal Autophagy Level of Rats

After all behavior experiments were completed, two rats of each group were anesthetized with the configured pentobarbital sodium. The brain tissues of rats were separated quickly. The blood clot and impurities on the surface of the brain tissue of the isolated rat were gently rinsed with ice physiological saline, and then the brain tissue was separated in the ice water. The hippocampal tissue was exposed to 5 pieces of hippocampal tissue of about 1–2 mm^3^ and fixed overnight at 4°C refrigerator. The following day, the sample was sent to Shanghai Zhicheng Biological Technology Co., Ltd., for sample embedding (after cleaning with 0.1 M phosphoric acid buffer for three times, then using 1% osmium acid at 4°C to fix 3 h). Then the buffer solution was cleaned three times, the ethanol was dehydrated, the epoxy propane was replaced, and the Spurr resin was soaked and buried. Finally it was polymerized at 70°C. The embedded blocks of different materials were sliced on the ultrathin section machine. The thickness of ultrathin sections was 70 nm, stained with Uranyl acetate and lead citrate, and observed and photographed under transmission electron microscope.

### Statistical Analysis

Statistical analysis was performed by SPSS soft version 20.0 (Chicago, IL, United States). Between-group effects on escape latency in the MWM task was analyzed by repeated-measures analysis of two-way ANOVA with group and time as the factors followed by the least significant difference (LSD) *post hoc* test. Statistical analyses of other parameters were carried out using one-way ANOVA followed by the LSD *post hoc* test. The data are expressed as the mean ± standard error of the mean (SEM), and *P* < 0.05 was considered statistically significant.

## Results

### H_2_S Improves the Cognitive Function of B2M-Treated Rats in Y-Maze Test

**FIGURE 2:**
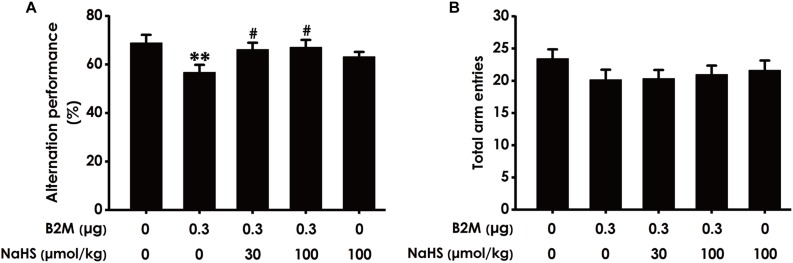
Effect of H_2_S on B2M-induced cognitive impairments of rats in Y-maze test. Seven days after intracerebroventricular administration of B2M (0.3 μg), the rats were submitted to the Y-maze test. The alternation performance **(A)** and the total arm entries **(B)** of rats in each group were recorded. Values were represented as mean ± SEM (*n* = 8–12); *^∗∗^P* < 0.01, vs. control group; *^#^P* < 0.05, vs. B2M-treated alone group.

To investigate whether H_2_S mitigates the cognitive dysfunction of B2M-exposed rats, we examined the cognitive function of rats using the Y-maze test. As shown in Figure 2A, the correct rate of alternation in B2M-exposed rats was significantly lower than that in the control group, indicated that intracerebroventricular injection of B2M leads to impairment in the learning and memory of rats. However, treatment with NaHS (30 or 100 μmol/kg/d, i.p.) significantly increased the correct rate of alternation in the B2M-exposed rats (Figure 2A). In addition, the total times of rats entering each arm in the five groups was not statistically significant (Figure 2B). These results showed that H_2_S improves the cognitive ability of B2M-exposed rats.

### H_2_S Ameliorates the Cognitive Dysfunction of B2M-Exposed Rats in NOR Test

**FIGURE 3:**
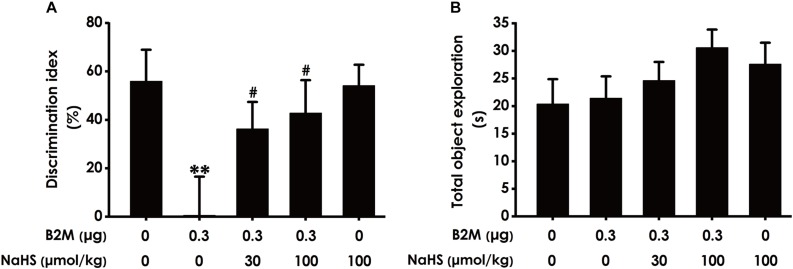
Effect of H_2_S on B2M-induced cognitive impairments of rats in Novel object recognition test. After the Y-maze test, the rats were submitted to the novel object recognition test. The discrimination index **(A)** and the total exploration time **(B)** of rats in each group were recorded. Values were represented as mean ± SEM (*n* = 8–12); *^∗∗^P* < 0.01, vs. control group; *^#^P* < 0.05, vs. B2M-treated alone group.

To further investigate whether H_2_S ameliorates the cognitive impairment in B2M-exposed rats, we also examined the cognitive function of rats using the NOR test. As shown in Figure 3A, the discrimination index in B2M-exposed rats was significantly decreased compared with the control group. However, NaHS (30 or 100 μmol/kg/d, i.p.) significantly increased the discrimination index of B2M-exposed rats. In addition, the total exploration time among these five groups had no significant difference (Figure 3B). Taken together, these data also suggested that H_2_S reverses the cognitive impairment induced by B2M.

### H_2_S Enhances Spatial Learning and Memory of B2M-Treated Rats in MWM Test

**FIGURE 4:**
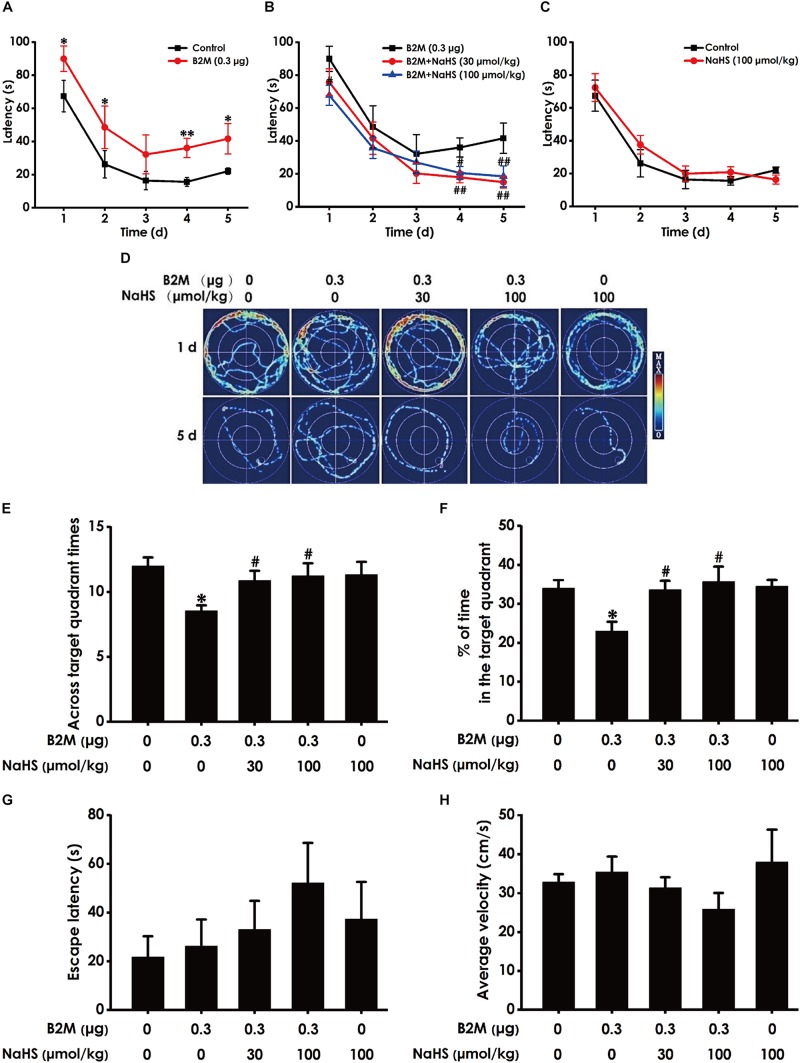
Effect of H_2_S on B2M-induced cognitive impairments of rats in Morris water maze test. After finishing NOR Test, the rats were tested by the Morris water maze test. Rats were submitted to acquisition phase of an invisible platform placed in a fixed location (target quadrant) with four times swim per day during 5 days. After finishing the acquisition phase, rats were submitted by the probe trail without platform, and then the visible platform test with platform. The latency time **(A–C)** and the swimming tracks **(D)** of rats searching for the underwater platform at the 1st to 5th training days were recorded. The times that rats crossed target quadrant **(E)** and the percentage of time spent in the target quadrant **(F)** in the probe trail were analyzed. The latency to reach the platform **(G)** and the average velocity of swimming **(H)** in the visible platform test were also recorded, respectively. Values were represented as mean ± SEM (*n* = 8–12); ^∗^*P* < 0.05, ^∗∗^*P* < 0.01, vs. control group; *^#^P* < 0.05, *^##^P* < 0.01, vs. B2M-treated alone group.

We also used the MWM test to investigate the protective role of H_2_S in the cognitive dysfunction of B2M-treated rats. The latency to find the platform in the acquisition phase is shown in Figures 4A–C. All five groups during the five training days exhibited a decrease in the escape latency (Figures 4A–C). B2M-treated alone rats exhibited significantly longer in escape latency in the 1st, 2nd, 4th, and 5th training day compared with the control group (Figure 4A), which implies a significant impairment of spatial learning in B2M-exposed rats. However, treatment with NaHS (30 or 100 μmol/kg/d, i.p.) significantly decreased the escape latency of B2M-treated alone rats in the 4th and 5th training day (Figure 4B). The escape latency in NaHS-treated alone rats was not significantly different from the control group (Figures 4C). Figure 4D shows the representative swimming tracks of rats searching for the underwater platform on the 1st and 5th training days. On the 1st training day, there was no difference of the distance in searching for the hidden platform among these five groups (Figure 4D). On the 5th training day, compared with the control group, B2M-treated alone rats exhibited a significant increase in the swimming distance, while the rats cotreated with NaHS (30 or 100 μmol/kg/d, i.p.) and B2M showed a significant decrease in the swimming distance compared with the B2M-treated alone group (Figure 4D).

In the probe phase, B2M-treated alone rats showed impaired memory, as evidenced by their significant decreases in the times of crossing the target quadrant (Figure 4E) and the time spent in the target quadrant (Figure 4F). However, NaHS (30 or 100 μmol/kg/d, i.p.) significantly increased the times that B2M-treated alone rats crossed the target quadrant (Figure 4E) and the time that B2M-treated alone rats spent in the target quadrant (Figure 4F). Together, these data suggested that H_2_S reverses the impairment in spatial learning and memory induced by B2M.

After the probe phase, visible platform test is performed to examine the escape latency and average swimming speed of rats to exclude the possibility that is caused by the changes of vision and motor ability in the rats. There was no statistical difference in the escape latencies (Figure 4G) among all five groups in the visible platform test, and there was no significant difference in swimming speed (Figure 4H), which reflects that different vision or motor ability of rats does not contribute to the alterations of all indicators in probe trial.

### H_2_S Reverses B2M–Inhibited Autophagic Flux in the Hippocampus of Rats

**FIGURE 5:**
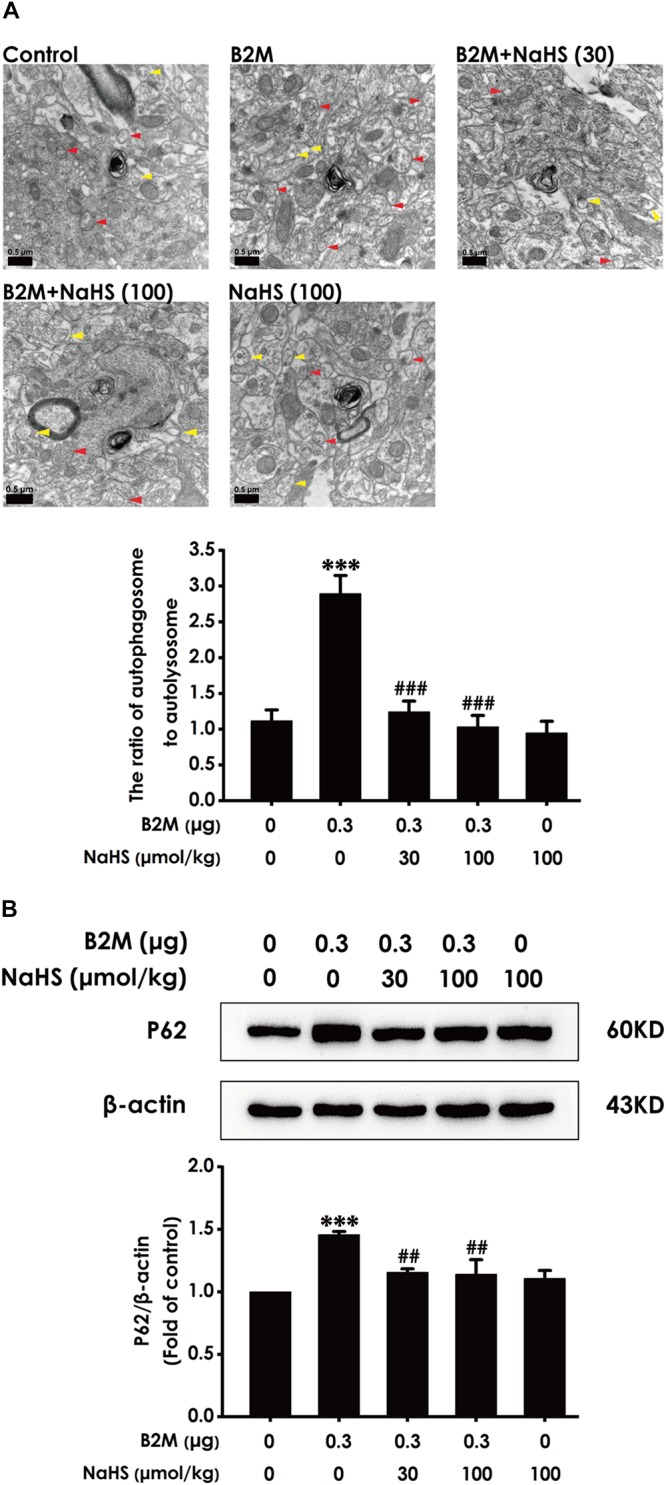
Effect of H_2_S on the hippocampal autophagic flux in B2M-exposed rats. After all behavioral experiments completed, the autophagy-associated markers in hippocampus were detected. **(A)** The number of autophagosome (Red arrowhead) and autolysosome (Yellow arrowhead) in the hippocampus was observed under transmission electron microscopy. **(B)** The expression of p62 was detected by Western blot analysis. Values were represented as mean ± SEM (*n* = 3); ^∗∗∗^*P* < 0.001, vs. control group; ^##^*P* < 0.01, ^###^*P* < 0.001 vs. B2M-treated alone group.

To investigate whether H_2_S-improved cognitive function in B2M-exposed rats is involved in the regulation of hippocampal autophagic flux, the number of autophagosome and autolysosome and the expression of p62 protein in the hippocampus were observed, respectively. Compared with the control group, the ratio of autophagosome to autolysosome (Figure 5A) and the expression level of p62 protein (Figure 5B) in B2M-exposed rats were significantly increased (*P* < 0.001), indicating that the hippocampal autophagic flux in B2M-exposed rats is blocked. After treatment with NaHS (30, 100 μmol/kg, i.p.), the ratio of autophagosome to autolysosome (Figure 5A) and the expression of p62 protein (Figure 5B) in the hippocampus of B2M-exposed rats were significantly decreased (*P* < 0.001), indicating that H_2_S restores the autophagic flux in the hippocampus of B2M-exposed rats.

## Discussion

The intentions of our study were to investigate the role of H_2_S in B2M-induced cognitive dysfunction and the underlying mechanisms. Our findings were as follows: (1) H_2_S attenuated cognitive dysfunction induced by B2M in rats; (2) H_2_S reversed the inhibition of autophagic flux in the hippocampus of rats treated with B2M. These results demonstrated that H_2_S ameliorates cognitive impairments induced by B2M, which is related to the recovery of hippocampal autophagic flux.

Exposure of B2M has certain toxicity in nervous system of human beings, recognized as a useful marker in early diagnosis and monitoring of brain metastasis in malignant disease (Alsobhi et al., 2007; Hansen et al., 2009). Research has shown that B2M, a systemic pro-aging factor, negatively regulates neurogenesis in the hippocampus (Smith et al., 2015). The interference with hippocampal neurogenesis is an important pathophysiological mechanism of cognitive dysfunction. Moreover, B2M plays an important role in synaptic plasticity and development of the mammalian nervous system (Lyupina et al., 2013). Clinical studies have reported that the level of B2M is elevated in cerebrospinal fluid of AD patients and HIV related dementia patients (Mcarthur et al., 1992; Carrette et al., 2003). Thus, these findings lead us to guess how the relationship is between B2M and cognitive impairments. In our present study, B2M-exposed rats showed cognitive defects in Y-maze test, NOR test, and MWM test. Our results are in agreement with the research that the elevation of B2M in the hippocampus is associated tightly with the dysfunction of learning and memory (Starkey et al., 2012). Just as B2M is an imperative negative factor in the nervous system of human beings, it is of certain scientific value to explore how to prevent and treat the damage to the cognitive function caused by B2M.

Although H_2_S is an acknowledged toxic gas (Attene-Ramos et al., 2006), it has been recognized as a major biological messenger with in-depth research nearly two decades (Kimura et al., 2005). H_2_S have been discovered certain physiological concentration in the brain tissues of mice (Koike et al., 2017). Additionally, there is evidence to indicate that the toxic effect of H_2_S may be due to its high concentration (Su and Wong, 2017). Accumulating evidences confirmed that H_2_S produces antioxidant, anti-inflammatory, and antiapoptotic effects, which have relevance to the protective role of H_2_S in neurodegenerative diseases (Hideo, 2002; Li-Fang et al., 2011; Hideo et al., 2012). Moreover, it has been revealed that H_2_S facilitates long-term potentiation (LTP) and regulates intracellular calcium levels in the hippocampus, which are important processes in learning and memory (Nagpure and Bian, 2015). Previous works have demonstrated that H_2_S reverses learning and memory deficits of rats induced by homocysteine (Wei et al., 2014) and has a protective effect on beta-amyloid associated spatial memory impairments (Xuan et al., 2012). Hence, it has significant scientific value for us to elucidate the beneficial role of H_2_S in B2M-induced deficit of learning and memory function.

In this work, we found that administration of NaHS resulted in a conspicuous increase in the alternation performance in Y-maze test, a profound increase in the discrimination index in NOR test, and a significant increase in the crossing numbers and spending time in the target quadrant in WMW test in the B2M-exposed rats, suggesting that H_2_S alleviates the cognitive defects of B2M-exposed rats. Intriguingly, these results of three kinds of animal behaviors contribute to come up with this assumption that H_2_S may exhibit its protective effect in a concentration-dependent manner. In addition, it has been reported that H_2_S ameliorates cognitive dysfunction in streptozotocin-induced diabetic rats (Zou et al., 2017). Taken together, it is reasonable to believe that H_2_S prevents B2M-exposed rats from learning and memory impairments. More importantly, these findings also strengthen the importance of H_2_S in the aspect of improving cognitive function, and may provide a target for therapy of cognitive disorders.

Autophagy is the major intracellular degradation system by which cytoplasmic materials are delivered and degraded in the lysosome to maintain cellular renovation and homeostasis (Mizushima and Komatsu, 2011). Similarly to cell differentiation and death, autophagy is disturbed in multiple diseases, in which excessive or deficient autophagy may contribute to pathogenesis (Kroemer, 2015). Autophagic flux is a dynamic process in which cell substances are transferred to lysosomes for degradation and recycling (Levy et al., 2017). The low level of autophagy causes organelle and synaptic plasticity damage, leading to AD, PD and other neurodegenerative disease (Nixon, 2006). It has been reported that the inhibition of autophagic flux induces postsynaptic dysfunction and impairs memory function (Wang et al., 2019). Likewise, promoting autophagic flux could enhance cognitive performance (Puigoriolillamola et al., 2018). So we speculated that inhibited autophagic flux in hippocampus is inseparable from cognitive impairments of B2M-exposed rats. In this study, we found that the ratio of autophagosome to autolysosome and the expression of p62 protein showed an increasing trend in B2M-treated rats. These data demonstrated that B2M blocked the autophagic flux in the hippocampus of rats. Notably, the treatment of NaHS significantly reduced the ratio of autophagosome to autolysosome and the expression of p62 protein in B2M-treated rats, indicating that H_2_S reverses the inhibitory effect of B2M on autophagic flux in hippocampus. Interestingly, previous studies showed that H_2_S activates the autophagic flux in liver to reduce serum triglyceride level (Sun et al., 2015). In the colon cancer model, H_2_S regulates AMPK/mTOR pathway to induce protective autophagy (Wu et al., 2012). These previous studies provided a reasonable and strong evidence for our present results. Collectively, these results suggest that the recovery of hippocampal autophagic flux is an important mechanism involved in H_2_S antagonizing B2M-induced cognitive defects.

In summary, the present work has demonstrated that H_2_S improves the cognitive function and rescues the blockade of hippocampal autophagic flux in the B2M-exposed rats. Our data suggest that H_2_S reverses B2M-caused cognitive dysfunction by recovery of hippocampal autophagic flux. These findings establish the basis of our further study on the protective role of H_2_S through autophagy pathway and open a novel perspective that H_2_S might be a potential agent for protection against the cognitive impairment induced by B2M.

## Data Availability Statement

All datasets generated for this study are included in the manuscript/supplementary files.

## Ethics Statement

The animal study was reviewed and approved by the Animal Use and Protection Committee of University of South China.

## Author Contributions

X-QT and WZ contributed conception and design of the study. X-QT and PZ guided and supervised the study. S-MC, Y-LY, DZ, Y-YT, and XK performed the experiments. S-MC, Y-LY, and DZ analyzed the data of the experiments. S-MC and Y-LY wrote the manuscript. All authors contributed to manuscript revision, read and approved the submitted version.

## Conflict of Interest

The authors declare that the research was conducted in the absence of any commercial or financial relationships that could be construed as a potential conflict of interest.
